# Knotted proteins: A tangled tale of Structural Biology

**DOI:** 10.1016/j.csbj.2015.08.003

**Published:** 2015-08-19

**Authors:** Patrícia F.N. Faísca

**Affiliations:** Departament of Physics and BioISI—Biosystems & Integrative Sciences Institute, Faculdade de Ciências, Universidade de Lisboa, Campo Grande, Lisboa, Portugal

**Keywords:** Knotted proteins, Protein folding, Molecular simulations, Kinetic stability, Protein evolution

## Abstract

Knotted proteins have their native structures arranged in the form of an open knot. In the last ten years researchers have been making significant efforts to reveal their folding mechanism and understand which functional advantage(s) knots convey to their carriers. Molecular simulations have been playing a fundamental role in this endeavor, and early computational predictions about the knotting mechanism have just been confirmed in wet lab experiments. Here we review a collection of simulation results that allow outlining the current status of the field of knotted proteins, and discuss directions for future research.

## 1 Introduction

Proteins are the essence of life, playing crucial roles in virtually every biological process. Amongst many other functions, proteins drive and control our metabolism, protect us against viruses and bacteria, and allow us to breathe, to move and to see. In order to work properly, these extraordinary nanorobots, formed by many thousands of atoms, must acquire a specific biologically functional structure—the native state—through the process of protein folding. Typically, the native state coincides with the protein's tertiary structure, which results from the three-dimensional packing of the secondary structural elements (namely, alpha-helices and beta-strands). In a peculiar class of proteins, the so-called knotted proteins, the native state embeds a knot.

**Fig. 1:**
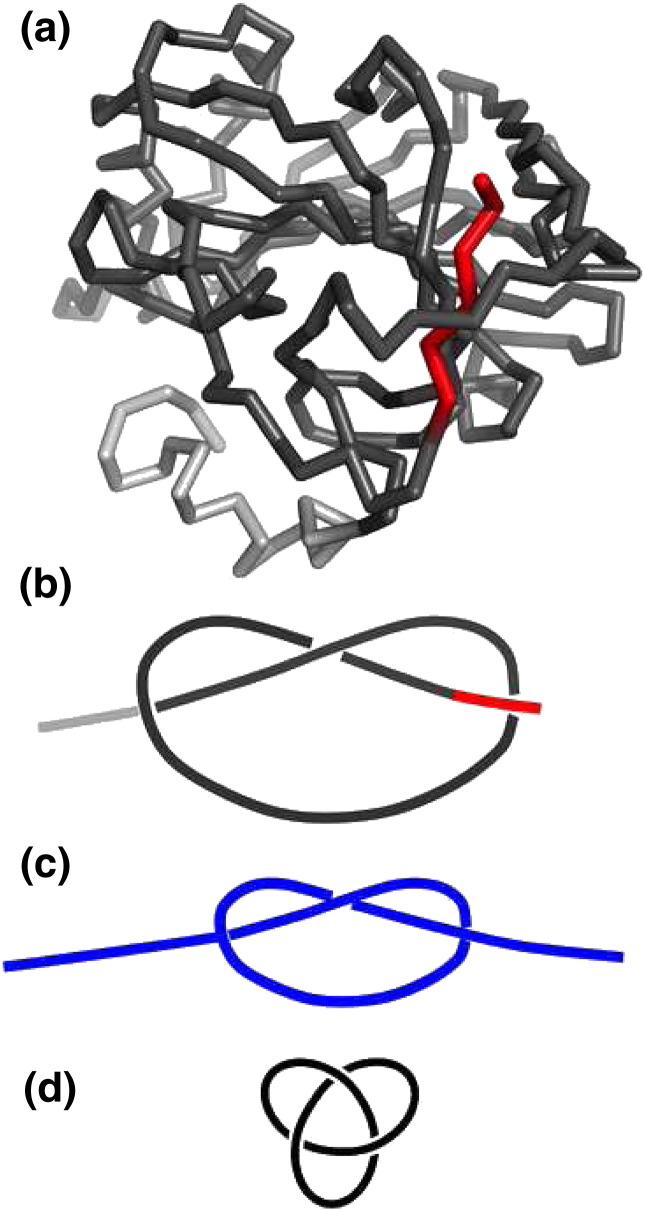
Ribbon representation (a) and planar diagrammatic representation (b) of the native structure of CAB (PDB ID: 2cab) illustrating the shallowness of the open trefoil knot. In both (a) and (b) the minimal segment that contains the knot—the knotted core is colored dark grey. It is enough to remove four residues from the carboxy-terminus (highlighted in red) to untangle the native structure. The carboxy-terminus is the shortest knot tail, while the N-terminus is the longest knot tail. Panel (c) represents a hypothetical planar diagrammatic representation of a deep trefoil knot with significantly more extended knot tails. In this case it is necessary to remove many residues from one of the two chain ends to untangle the protein. If the two chain ends are connected the physical knot gives rise to a topological trefoil knot as shown in (d). (For interpretation of the references to color in this figure legend, the reader is referred to the web version of this article.)

The first survey of the Protein Data Bank (PDB) that specifically investigated the occurrence of knots in proteins was performed by Mansfield in 1994. Out of the 400 entries analyzed only one, that corresponds to protein carbonic anhydrase B (PDB ID: 2cab), was found to be knotted [1] (Fig. 1a). It should be mentioned that while the discovery of a knot in CAB is generally attributed to Mansfield, the first researcher to report the existence of a knot in CAB was actually Richardson in 1977 [2]. Mansfield noticed that the knot in CAB is ‘incipient’ or ‘loosely formed’ since it is sufficient to remove a few residues from one terminus to untangle the protein (Fig. 1b). Using current terminology such protein knot classifies as shallow [3]. Given the shallowness of the knot found in CAB, Mansfield's investigation raised some skepticism regarding the existence of knotted proteins.

Indeed, in rigor, protein knots are not mathematical knots because the protein backbone does not form a closed curve in space [1,4] (they should therefore be classified as open knots or physical entanglements). Therefore, the so-called knot invariants such as the crossing number (the least number of crossings that occur in any planar projection of the knot) or the Alexander polynomial [5], which are used to distinguish between different knots, cannot be rigorously determined for knots in proteins. However, since the vast majority of knotted proteins have their termini located close to the minimal convex surface enveloping the entire protein structure [4], these ends can be in many cases unambiguously connected with a external arc forming a closed loop [1,4] (Fig. 1c). Mansfield took advantage of this possibility and used the Koniaris–Muthukumar method [6] to find out that CAB embeds a trefoil (or 3_1_) knot, i.e., a knot for which it is possible to find a planar projection with three crossings, and no projection with less than three crossings (Fig. 1d).

In 2000 Taylor developed an alternative computer method that can be applied when the chain termini do not lie at the protein's surface. Given a conformation, the algorithm's purpose is to produce a reduced, smoothed and ‘topologically equivalent’ representation in which the knotted core, i.e., the minimal chain segment that contains the knot, is sufficiently far from both chain ends for the knot type to be well defined. In Taylor's method the chain ends are kept fixed (and unconnected) throughout the whole procedure. Taylor discovered a deep figure-eight (or 4_1_) knot (i.e. a knot for which there is no planar projection with less than four crossings) in protein acetohydroxy acid isomeroreductase (PDB ID: 1yvel) [7], which can only be removed if 70 residues are deleted from the carboxy-terminus or 245 residues are deleted from the amino-terminus. Because this knot is deep, Taylor's discovery represents a rather important contribution that actually boosted research in the field of knotted proteins.

**Fig. 2:**
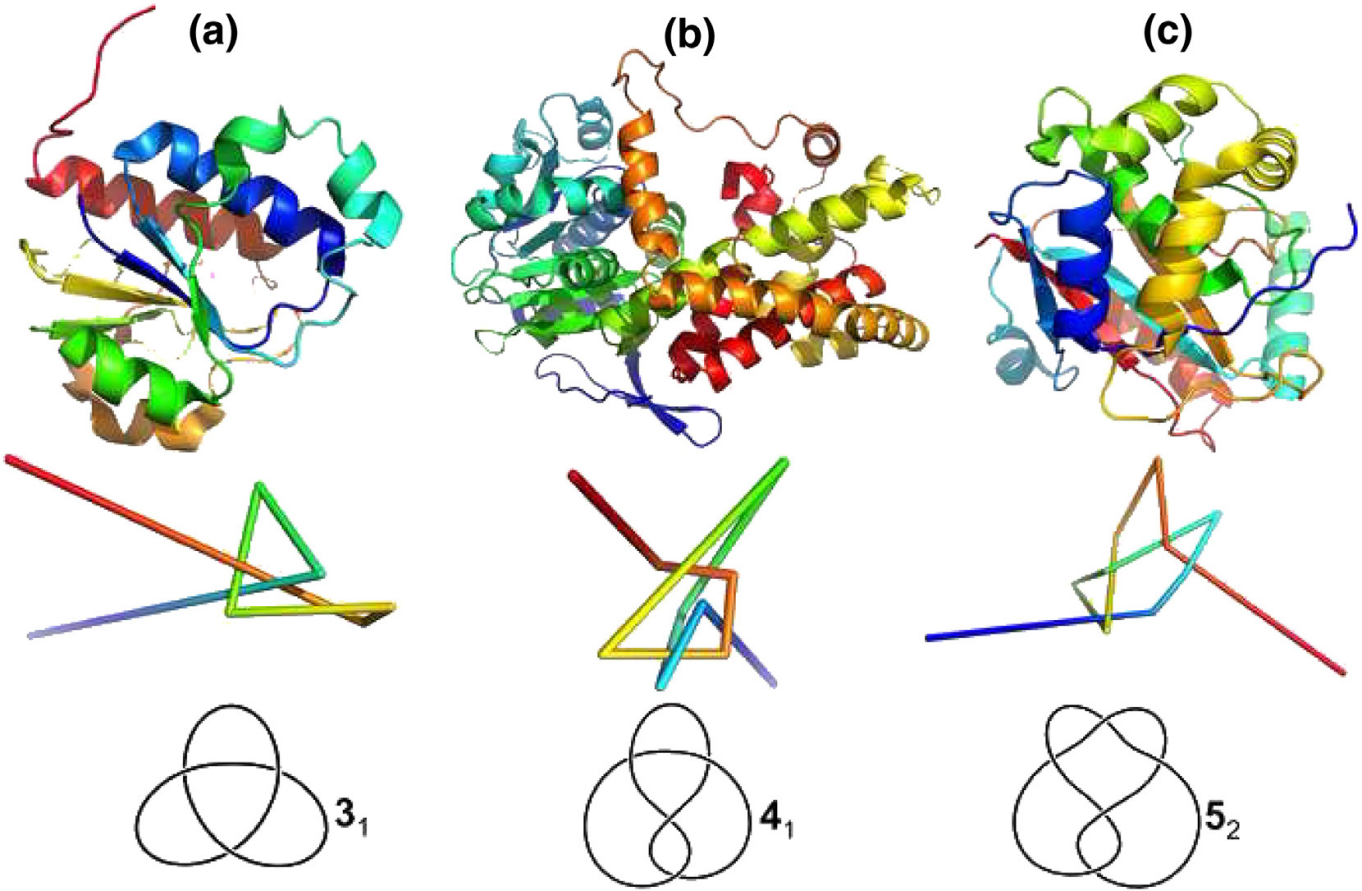
Cartoon representation of the native structure (top), reduced backbone representation obtained with the Taylor smoothing algorithm highlighting a open knot (middle) and corresponding topological knot (bottom) of proteins YibK (PDB ID: 1j85) (a), acetohydroxy acid isomeroreductase (PDB ID: 1yve) (b), and UCH-L3 (PDB ID: 1xd3) (c). The trefoil (or 3_1_), figure-eight (or 4_1_) and penta (or 5_2_) knots exhibit three, four and five crossings on a planar projection. The subscript 1 in 3_1_ (4_1_) stands for first knot with three (four) crossings and subscript 2 in 5_2_ stands for second knot with five crossings, according to standard knot tables The coordinates of the reduced representations were retrieved from http://knots.mit.edu/ and visualized with PyMol (The PyMOL Molecular Graphics System, Open-Source 1.5.x). The topological representations were produced with knotplot (http://www.knotplot.com/).

Taylor's algorithm is applied to a linear conformation and it was noticed that the final outcome may depend on the order of smoothing operations (i.e. if it starts at the N-terminus or at the C-terminus) [8]. Therefore, the initial conformation needs to be closed prior to the smoothing procedure through a closure method (reviewed in [9]). The combination of smoothing algorithms with closure methods and knot invariants provides a straightforward way to systematically scrutinize the universe of protein conformations deposited in the PDB in searching for different knots (Fig. 2).

**Fig. 3:**
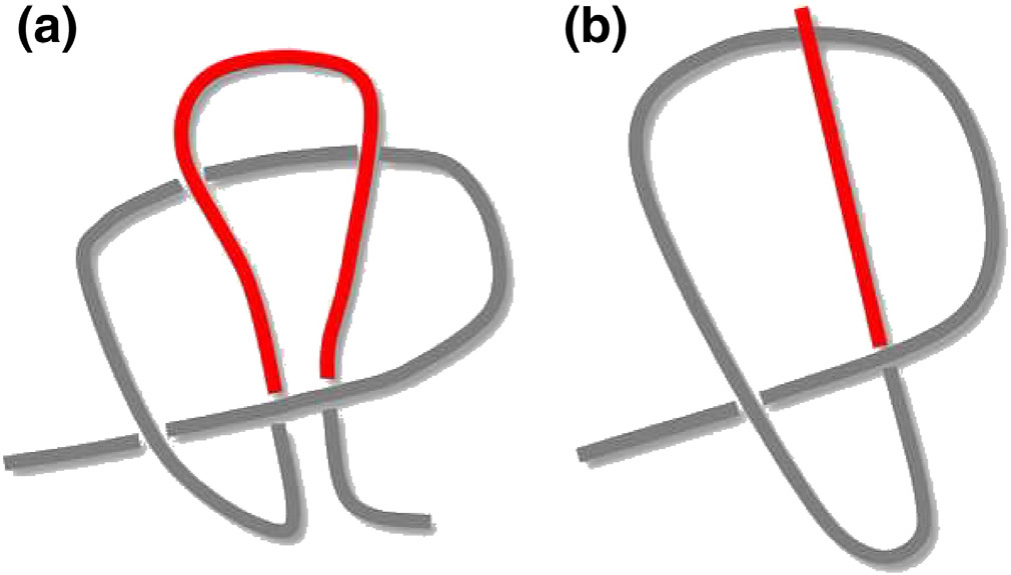
A slipknot (a) is a conformation in which one of the protein termini adopts a hairpin-like conformation (highlighted in red) that threads a loop formed by the remainder of the chain. A knotting mechanism based on slipknots has been proposed for some proteins. In alternative, the knotting step may occur via the threading of one of the termini through the knotting loop. (For interpretation of the references to color in this figure legend, the reader is referred to the web version of this article.)

An interesting variation amongst knotted conformations is that of the slipknot, originally identified by Yeates and co-workers [10]. A slipknot is a structure that when examined in its complete form is unknotted, but becomes knotted by deletion of suitable terminal segments because—just like in a shoelace—it is the arrangement of the terminal segment that unties the knot as the chain folds back upon itself close to one of its ends (Fig. 3a). The most recent survey of the PDB found 1150 tangled proteins (including slipknots) amongst 144,554 analyzed protein entries [11]. Tangled topologies, and knotted proteins in particular, thus represent a small fraction of the folding space represented by the PDB. There are many proteins with the 3_1_ knot in their native structure, some with the 4_1_ knot and only a few with the 5_2_ knot [12–14]. So far, the most complex knot type found in the PDB is the Stevedore's (or 6_1_) knot, which was detected in protein α-haloacid dehalogenase (PDB ID: 3bjx) [15]. Information on knotted proteins can be retrieved from databases that have been created along the years [13,16]. KnotProt is likely the most up-to-date collection of tangled structures that cover knotted proteins as well as slipknots [11].

Since Taylor firstly provided compelling evidence for the existence of deeply knotted proteins, a body of experimental and theoretical work has been developed that seeks to address two fundamental questions, 1) how do knotted proteins fold? and 2) what is the functional role of knots in proteins?

Understanding how *regular* proteins fold is considered one of the most challenging questions in science [17]. The existence of deeply knotted proteins, which represent extreme cases of topological complexity, raised the difficulty of the game play. Therefore, and perhaps not surprisingly, much of the work that has been developed to solve this particular folding puzzle has been focusing on the simpler trefoil knots, with a few studies looking at other knot types [15,18,19]. In particular, computer simulations based on a wide array of protein representations and sampling strategies [20–24], ranging from Monte Carlo simulations of lattice models [18,25,26] to Molecular Dynamics simulations of realistic force-fields [22,27], have been playing a decisive role in deciphering the folding mechanism of knotted proteins.

Here, we review a collection of computational results that provide a picture of current progress in this area. Although this article is focused on molecular simulation studies, we will also discuss experimental results to complement the theoretical views. To facilitate a critical analysis of the data provided by simulations we start by making a brief summary of the models and computational methodologies that are used to study protein folding, emphasizing their advantages and caveats. Subsequently, we discuss the physical reasons behind the rarity of knots in proteins. We proceed by presenting a series of results that convey the current views on their folding mechanism, and subsequently analyze the functional role of knots in proteins. Finally, we draw some concluding remarks and outline directions for future research in this new field of protein science.

### 1.1 Molecular simulation methods for protein folding

It is widely accepted that computer simulations have been playing a fundamental role in protein folding research. Since the 1990s, the use of Monte Carlo simulations of lattice models has been helping establishing the fundamental principles driving this remarkable biological process [28–33]. More recently, these models have been used to study aggregation and other biologically relevant phenomena involving proteins [34–38]. In a simple lattice model the protein is reduced to its backbone structure: amino acids are represented by beads that occupy the vertices of a (two or three dimensional) regular lattice and the peptide bond is reduced to sticks of uniform size (corresponding to the lattice spacing). Interactions between the amino acids can be modelled by the HP potential [39], that captures the hydrophobic effect by considering hydrophobic and hydrophilic amino acids only, by the sequence-based potential, which takes into account the heterogeneity of interactions resulting from the 20 amino acid alphabet by using the Miyazawa–Jernigan interaction matrix [40], or by the native-centric (or structure-based) Go potential, in which the interaction matrix is exclusively dictated by the native structure of the model protein, i.e., only native interactions contribute to protein energetics [41]. Lattice models are crude representations of real proteins that feature the fundamental ingredients of their polymeric nature. They are adequate to explore fundamental aspects of the folding process that do not depend on specific details of proteins, and computational efficiency allows evaluating folding thermodynamics and kinetics (including rates) with high accuracy.

To address the folding process of specific proteins researchers developed another class of models, which use an off-lattice representation of the protein (that can be either full-atomistic or restricted to C_α_ atoms) [42–49]. The folding space of off-lattice models is often explored with Monte Carlo (MC) methods or Molecular Dynamics (MD) schemes (discrete MD, Langevin etc). Off-lattice models are devoid of the severe restrictions imposed by the lattice, a disadvantage that, in particular, impairs a correct capturing of the conformational entropy [50]. In general, these off-lattice representations are combined with Go or Go-like interaction potentials. However, other structure-based models, based on more sophisticated intermolecular potentials, have been developed that incorporate important aspects of protein energetics (e.g. hydrogen bonding [51] and electrostatic interactions [23,52], just to mention a few examples) broadening the spectrum of the questions that can be tackled in the framework of simulations. A very interesting study by Holzgräfe and Wallin, combining a C_α_ representation with an interaction potential based on a three-letter amino acid alphabet, was recently developed to study the intriguing phenomena of protein fold switching [53].

At the top of hierarchical complexity one finds full atomistic representations combined with realistic force fields (e.g. AMBER and the GROMOS are popular choices among researchers), which are explored with classical MD simulations [54]. Apart from providing realistic energetics this approach allows one to simulate folding in explicit water. Its major advantage is the possibility to directly compare simulation data with data from in vitro experiments [55]. A fundamental problem of classical MD is the accuracy of the force fields. Another (less important) drawback is the need to consider very small time steps to integrate the equations of motion. This constraint imposes severe limitations on the total amount of simulation time and renders their systematic application to protein folding (and other dynamical processes involving large scale conformational changes) non-trivial. For this reason, smart sampling methods [56] and sophisticated distributed computing schemes [57,58] have been developed to conduct classical MD of protein folding, and novel algorithms and machine architectures have been created to execute MD simulations orders of magnitude faster than was previously possible [59]. A paradigmatic example of the latter is the ANTON machine developed by DE Shaw Research [60,61].

### 1.2 Why are knotted proteins rare?

Proteins (including knotted ones) fold into well-defined native structures. The specificity of the native structure results into an important difference between homopolymers (and DNA) and proteins: in proteins the same knot type is formed reversibly and reproducibly [62] in the same protein location [9] (homopolymers and DNA, on the other hand, collapse into non-specific ground states and the knot type and its location are, therefore, unspecific). Furthermore, as expected from polymer physics considerations, the formation of knots in homopolymers and DNA beyond a certain length is ubiquitous [63–65]. On the other hand, a recent analysis of the PDB revealed that the number of knotted proteins found so far is remarkably small (0.8% including slipknots [11]). Can we conclude from this observation alone that knotted proteins statistically rare? No. Indeed, in order to answer this question it is necessary to compare the frequency of knots in proteins with that observed in random heteropolymers of comparable length, compactness, and flexibility. In doing so, Grosberg and co-workers showed that knots in proteins are indeed statistically rare (and proteins are less knotted than expected), and further proposed that the rarity of knots in protein results from the *local* geometric features of proteins (e.g. subchain size and subchain interpenetration) that do not favor tangling of the backbone [66].

A very recent study based on a three-dimensional lattice representation, combined with the HP potential, reported a series of illuminating results that are in line with the idea that knots in proteins are indeed statistically rare. By conducting massive Wang-Landau Monte Carlo [67] simulations of the HP model, Virnau and co-workers were able to establish that proteinlike HP sequences strongly influence the degree of knottedness under conditions favoring the native state [68]. In particular, HP sequences that induce local structure (i.e. order) inside the hydrophobic core strongly inhibit entanglements and knots. Despite the rather large ground-state degeneracy of the HP model, the results obtained by Virnau and co-workers strongly support the view that knots in proteins are statistically rare because protein sequences impose local structural ordering.

Interestingly, a previous investigation that performed a systematic quantitative comparison of knotted and unknotted proteins in the PDB found that knotted proteins contain loop segments that are absent from sequence-homologous or structurally-similar unknotted proteins. The latter were identified as being “knot-promoting regions” since their removal results into unknotted conformations [69]. Therefore, while protein sequences in general disfavor knotted topologies, the sequences of knotted proteins appear to have evolved to encode specific structural features that drive, or at least facilitate, knotting.

Remarkably, knots are virtually absent in the RNA realm, with only three conformations (with ~ 3000 nucleotides) having been identified as deeply knotted amongst 6000 PDB entries analyzed in a recent survey [70]. While the reason for this observation remains unclear, it was suggested that a mechanism of co-transcriptional folding, favoring the formation of local helices, could rule out the occurrence of major entanglements in RNA, in line with the view that local order blocks knotting in biomolecules [70].

### 1.3 Knotting mechanism in off-lattice simulations

Even if knotted proteins are statistically rare, a complete understanding of the folding puzzle will not be achieved unless their folding mechanism(s) is solved [62]. A seminal study by Shakhnovich and co-workers explored the folding mechanism of protein YibK (PDB ID: 1j85) (Fig. 2a), which contains a deep trefoil knot in its C-terminal region [20]. Initially, the researchers conducted Langevin simulations of a C_α_ Go model that failed to correctly knot the protein. The authors reported that in order to achieve successful (and statistically significant) folding it was necessary to modify the native-centric Go potential by including a set of *specific* (i.e. sequence-dependent), weakly attractive non-native interactions. The analysis of 100 folding trajectories revealed that the *hybrid* Go potential was able to fold the protein (with 100% efficiency) by driving a knotting mechanism based on a threading movement of the C-terminus through a knotting loop formed by the remainder of the chain; a process similar to threading a line through the eye of a needle (Fig. 3b).

The fraction of formed native interactions, *Q*, is often considered a good reaction coordinate for folding whenever protein energetics is modeled by the Go potential [71–73]. In [20] two folding pathways were identified, one where the formation of the knot occurs late (when *Q* is ~ 0.8) and another where it forms earlier. The authors further conjectured that the non-native interactions could transiently transform the natively helical C-terminal region into a beta-sheet structure (which is dominated by non-local, long-range interactions) during the knotting step, in line with the idea that local ordering disfavors knotting.

A functional role for non-native interactions in the folding of protein AOTCase (PDB ID: 2 g68), which embeds a deep trefoil knot in its native structure, was later reported by Škrbić and co-workers based on MC simulations of a C_α_ model, with protein energetics incorporating non-native and electrostatic interactions [23]. In particular, the inspection of ~ 150 folding trajectories revealed that specific non-native interactions are important in promoting (or disfavoring) the formation of knots in the early stages of folding, and corroborated the importance of a knotting step based on a threading movement of the C-terminal part through a knotting loop (Fig. 3b).

An important study on YibK by Sulkowska and Onuchic, framed on a C_α_ Go model, reported a success rate of folding of 1–2% [21]. While the latter is not statistically significant, the analysis of ~ 10 successful folding trajectories allowed concluding that when folding is exclusively driven by native interactions the knotting step occurs through the formation of a folding intermediate with a slipknot (Fig. 3a) that forms typically late during folding (when *Q* ~ 0.8). According to this study, in the absence of non-native interactions, knotting via threading the C-terminus through the knotting loop (Fig. 3b) is still possible but it appears to be a rare event. Subsequent studies by the same group further emphasized the importance of slipknotted conformations in the folding of knotted proteins. In particular, knotting via slipknotting was also reported for MJ0366 (PDB ID: 2efv) the smallest knotted protein in the PDB (82 residues) in a MD study that combined a full atomistic representation of the protein with the Go potential [24]. Although threading the C-terminus is observed for MJ0366, slipknotting is the dominant knotting mechanism below the melting temperature *T_m_* (i.e. when the native state is thermodynamically stabilized relative to the denatured state) or when an extended C-terminal tail is added to the protein.

More recently, classical MD simulations in explicit water were conducted for protein MJ0366 in the ANTON super-computer. It was found that 5 (out of 15) folding simulations that started from slipknotted conformations were able to reach the native state through a knotting mechanism involving *similar* native contacts as those driving the knotting mechanics under the Go potential [27]. However, a previous MD study from Beccara and co-workers, which combined a realistic force field with a smart sampling scheme that allowed capturing 31 complete folding trajectories (in the absence of water), showed that when no structural constraint is placed on the starting conformations the knotting mechanism of protein MJ0366 occurs mostly (i.e. in 26 trajectories) via threading of the C-terminus through a knotting loop formed at an earlier stage [22]. Slipknotting was also observed, but only in three folding trajectories. It seems, though, that non-native interactions favor a knotting mechanism based on a threading movement of one of the protein's termini through a knotting loop formed by the remainder of the chain (Fig. 3b). On the other hand, native interactions appear to drive a tangling mechanism based on slipknots (Fig. 3a). Interestingly, a recent computational study based on unbiased MD simulations of a general homopolymer model predicted that the most likely knotting mechanism in homopolymers is based on the threading movement of the chain terminus through a knotting loop. Nevertheless, if the chain length exceeds 2000 monomers, a small but yet sizable fraction of knotting events proceeds via slipknotting [74]. This result suggests that chain length (which is a fundamental property of the polymer) can also contribute to balance the relative importance of the two knotting mechanisms identified so far in off-lattice simulations of protein folding.

The role of non-native interactions in protein folding has been explored by several authors, with many studies agreeing on a functional role played by these interactions [75–80], while others concluding they play essentially no active part [81]. A recent contribution by Best and co-workers, which analyzed a set of classical MD trajectories of nine proteins obtained by the Shaw group, concluded that non-native interactions are irrelevant to the mechanism of folding in most cases [82]. On the other hand, the current understanding of the folding process of knotted proteins clearly points out to a functional role of non-native interactions in the folding of knotted proteins. Concretely, the dominant knotting step is determined by non-native interactions depending on the type (stabilizing or destabilizing) and degree of their participation in folding energetics.

### 1.4 Knotting mechanism in lattice simulations

Contrary to what happens with off-lattice models it is straightforward to fold small, knotted proteins on-lattice with the Go potential. In our very first study of a small shallow trefoil knot we observed knotting from a precursor conformation with a slipknot [25]. However, a systematic analysis of the knotting step, based on extensive conformational clustering [83], and thousands of folding trajectories, revealed that it occurs predominantly via threading of one of the terminuses through a knotting loop formed by the remainder of the chain [18,26,84]. By tethering each terminal bead to a chemically inert plane we further concluded that knotting occurs predominantly via threading of the chain terminus that stands nearer the knotted core (i.e. the shortest knot tail), a process that costs less entropy than threading the longer tail [84]. Threading the shortest tail was also observed in folding simulations of a knotted protein designed in the Yeates Lab (PDB ID: 2ouf.pdb) [85], and, more recently, in vitro experiments of protein YibK [86]. A similar knotting mechanism was also found for a lattice protein embedding a shallow 5_2_ knot [18].

**Fig. 4:**
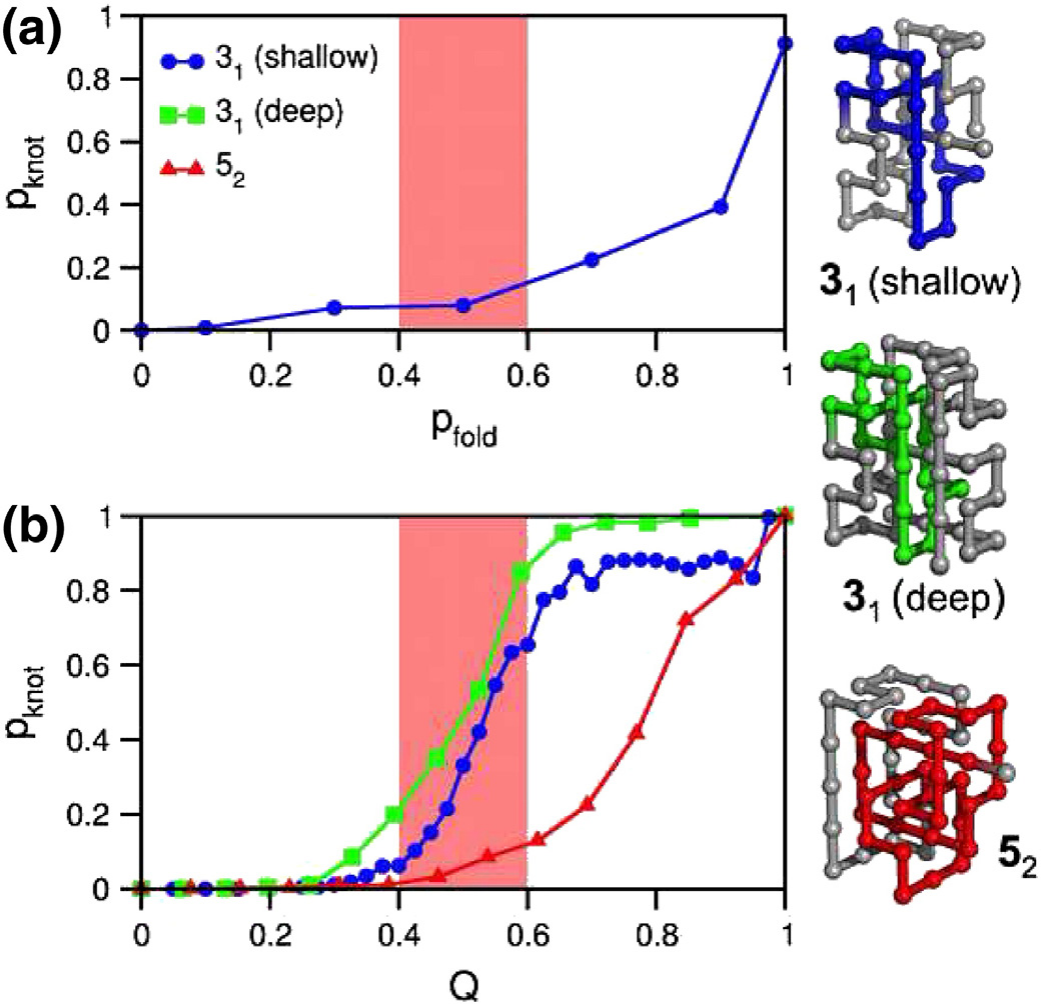
Knotting probability *p*_*knot*,_ as a function of the folding probability, *p_fold_* (a) and as a function of fraction of formed native contacts, *Q* (b) in three lattice Go proteins: a shallow trefoil knot, a deep trefoil obtained from the first by extending the knot tails, and a shallow 5_2_ knot. In the three dimensional structures the knotted core is highlighted. The three model systems fold with a thermodynamic two-state transition (data not shown) and the transition state ensemble comprises conformations in the region highlighted in pink. For both shallow and deep trefoils *p_knot_* shows a sigmoidal dependence on *Q* and there is a non-negligible probability for knotting and folding to occur concomitantly. For the more complex knot type knotting occurs in highly native-like conformations (*Q* > 0.8). *p_knot_* is the fraction of knotted conformations in very large ensembles of conformations (with fraction of native contacts *Q* or folding probability *p_fold_*) that are extracted from an equilibrium distribution at the temperature of interest. Details on the calculation of *p_fold_* can be found in [25].

The folding probability, *p_fold_* (i.e. the probability that a conformation folds before it unfolds), measures how kinetically close a conformation is from the native one [87]. The stage of the folding process at which knotting is more likely to occur can be inferred with high accuracy from the dependence of the knotting probability, *p*_*kno*t_, on the reaction coordinate *p_fold_* [25]. We found that knotting of our shallow trefoil knot occurs exceedingly late in folding, in conformations with *p_knot_* > 0.7 (Fig. 4a). An alternative, less computationally expensive measure of knotting progress, often used in off-lattice simulations, employs the fraction of native contacts, *Q*. It should be stressed that *Q* probes any knotted topology (including malformed knots) while *p_fold_* probes mostly natively knotted topologies. Accordingly, the knotting probability is always higher when folding progress is monitored with *Q*. Indeed, for the knot 3_1_, *p_knot_* shows a sigmoidal dependence on *Q* at *T_m_*, increasing sharply from ~ 0 to ~ 0.5 when *Q* = 0.5 [84] (Fig. 4b). A qualitatively similar behavior was reported in [85] for protein 2ouf, which also contains a shallow trefoil. However, for the knot 5_2_ the threading step occurs much later, when the fraction of native contacts is larger than 0.9 [18] (Fig. 4b). This different timing for the knotting step is due to the larger knotting loop of the knot 5_2_ (representing 60% of the chain length against 24% for the knot 3_1_) that must form earlier and be in place for threading to occur. Therefore, independently of how folding is probed, and which model representation is adopted, results obtained so far by different groups [20,21,25], including experimental ones [86,88,89], point to a mechanism where the knotting step occurs late to very late during folding.

Intermediate states are partially folded (i.e. neither completely folded nor completely unfolded) conformations that may facilitate or hamper folding. In the former case the formation of intermediate species precedes that of the native structure (i.e. the intermediate state is en route to folding), while in the latter the protein gets trapped in some malformed conformation which delays (or even blocks) the formation of the native fold. Such non-productive conformation forms because of stabilizing non-native interactions, or as a result of the complexity of the native sate, which leads to topologically trapped conformations. Intermediate states are elusive species in the folding space of small (~ 150 amino acids) proteins which fold preferentially with two-state kinetics [90] in smooth free energy landscapes. However, in the case of knotted proteins, even systems of small size such as protein MJ0366, the peculiar topological complexity of their native state leads not only to the formation of topological traps but also to the formation of intermediate states which are enroute to folding [20,24,84]. Intermediate states (productive and non-productive) are therefore recurrent species in the folding landscapes of tangled proteins.

### 1.5 Knotting mechanism, backtracking and structural mutations

**Fig. 5:**
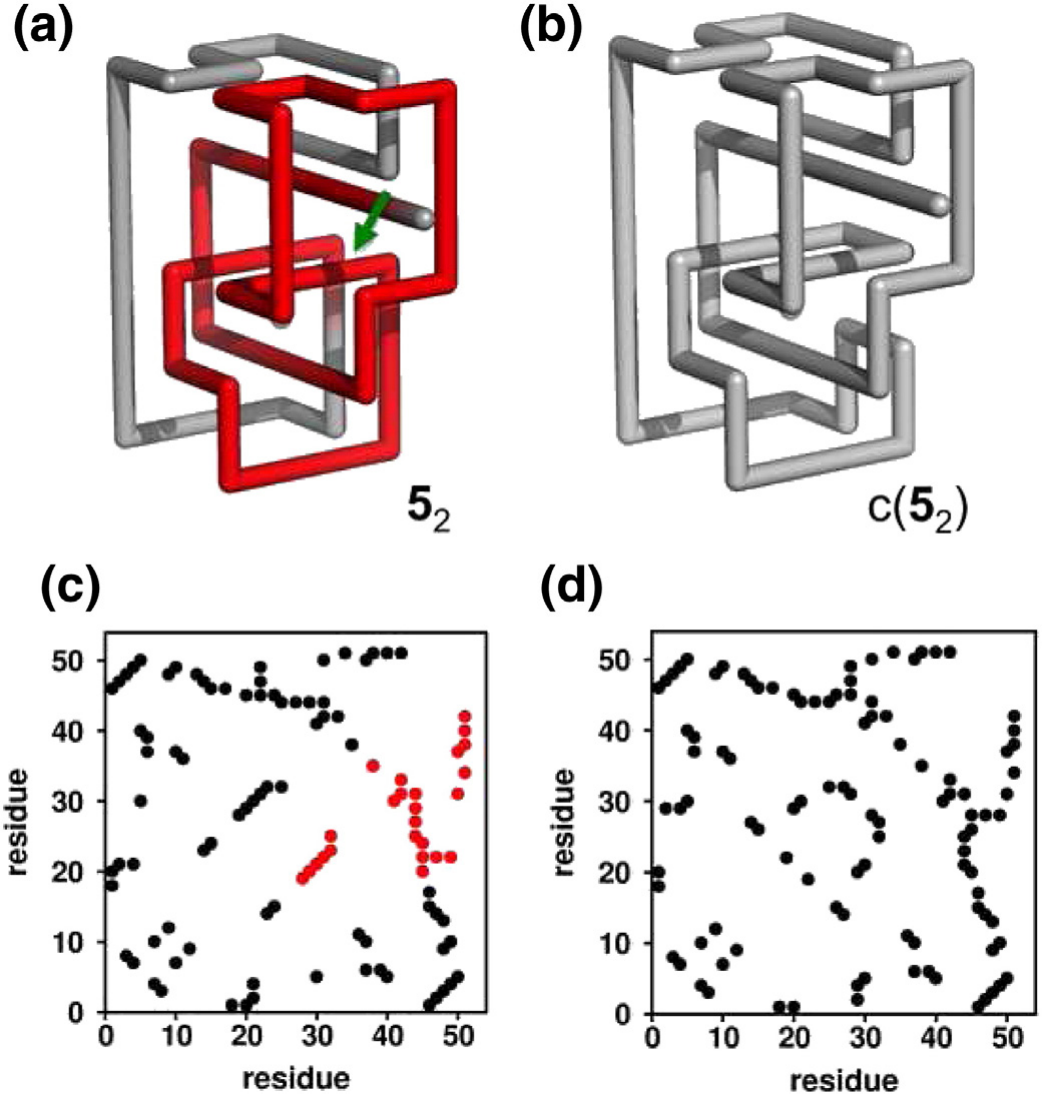
Three-dimensional representation of the lattice protein with a 5_2_ knot embedded in its native structure (a) and a unknotted control conformation with a remarkably similar structure (b) that was obtained from (a) by minimally changing the connectivity of the chain (in the site indicated by the arrow). The contact maps, showing the total number of native contacts, also highlight the similarity between the 5_2_ knot (c) and its control system (d). The knotted core and native contacts establishing between the knotted core residues are highlighted in red in the three-dimensional structure and contact map, respectively.

An important advantage of lattice models is the ability to easily create an unknotted conformation by minimally modifying the backbone connectivity of a knotted template (Fig. 5). The conformation thus created can be used as a control system and folding behavior of the two model proteins can be directly compared to determine the effects of knots on folding properties (e.g. folding rate).

**Fig. 6:**
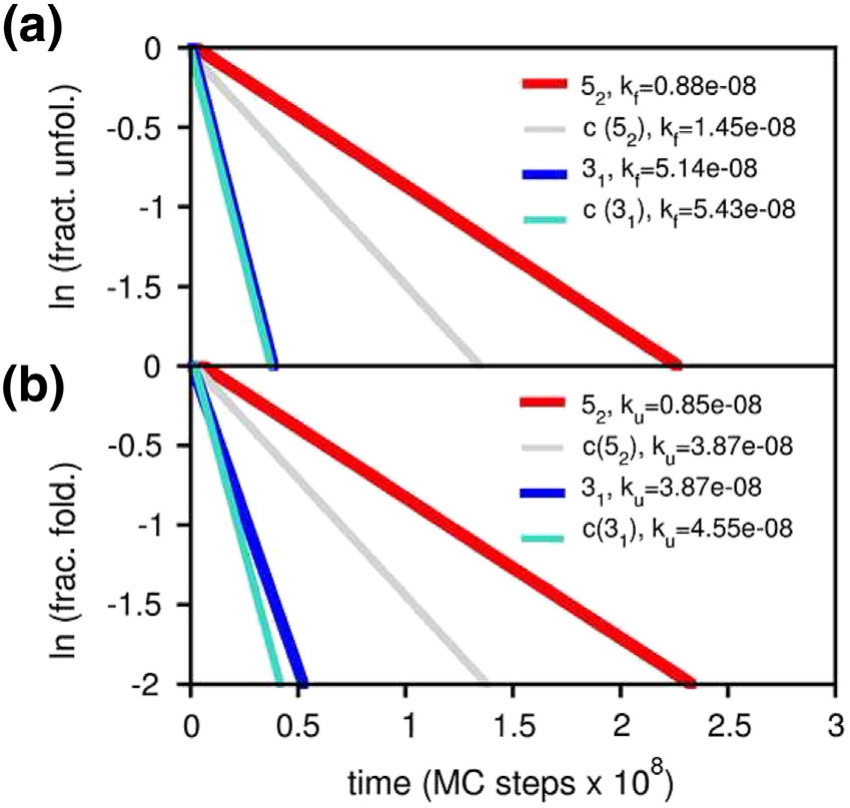
Folding (a) and unfolding (b) kinetics for the lattice 5_2_ knot and the lattice (shallow) trefoil. The folding (*k_f_*) and unfolding (*k_u_*) rates are given by the slope of the regression lines. Both knot types fold and unfold much slower than their unknotted control systems. However, the difference in folding and unfolding rates is much larger for the more complex knot type.

By performing highly accurate measurements of the folding rate (based on ~ 2000 MC trajectories), we observed that the folding of knotted lattices is always slower than that of its unknotted counterparts [26,84], in line with an earlier experimental observation [91], and predictions from off-lattice simulations [24,85]. Furthermore, we noticed that folding becomes remarkably slower as the complexity of the knot increases [18] (Fig. 6a).

In part, the slow folding rates of knotted proteins are due to the phenomenon of backtracking, i.e., the breaking and re-establishment of specific native contacts [21,92]. Indeed, since knotted proteins fold through an ordered process [24], backtracking will necessarily occur if folding does not start from the “right” conformation, or if it follows an incorrect order (or sequence) of events leading to malformed knots and other topologically trapped conformations [24,26,85], which results in large folding times.

To probe the importance of backtracking in the folding of knotted proteins we proposed a special type of mutation termed structural mutation (SM) [18]. SMs disrupt native interactions that establish between residues that are located on the threading terminus or within the knotting loop (i.e. in structural elements that are key for knotting), but do not play a role in the energetic stabilization (i.e. nucleation) of the transition state [93] in proteins with a two-state folding transition. SMs are expected to increase the folding rate because they should decrease the probability of occurrence of topological bottlenecks resulting from a premature establishment of the corresponding wild-type (WT) interactions in loosely folded conformations. In other words, SMs should decrease backtracking.

The results obtained on lattice proteins are actually in line with this rationale since a significant increase in folding rate for both knot types is observed upon performing SMs [18]. Moreover, the enhancement is clearly larger for the knotted protein with the 5_2_ knot suggesting, perhaps not surprisingly, that backtracking is more prominent in knots of higher complexity [18]. Interestingly, we also observed that SMs accelerate the unfolding process considerably, presumably because they perturb the structural stability of the knotting loop. A less stable knotting loop should facilitate the untangling (and further unfolding) of partially folded, or misfolded conformations. It would be very interesting to explore through in vitro experiments the importance of structural mutations as probes of backtracking.

### 1.6 Knotting mechanism in vitro and in vivo

Experimental work on knotted proteins has been essentially developed in the Yeates [3,10,91,94] and in the Jackson laboratories. The Jackson Lab, in particular, has been playing a leading role in establishing the folding mechanism of knotted proteins. With one exception [19], experimental work has been focusing on knotted trefoils YibK (Fig. 2a) and YbeA (PDB ID: 1ns5) [95–98]. An important result from Jackson's experiments is the observation that the denatured state of YibK and YbeA remains trapped in a knotted topology, even in high concentrations of chemical denaturant [99]. This finding is of critical importance as it implies that the subsequent refolding does not start from an unknotted conformation (as it does in simulations), and, therefore, the interpretation of relevant folding properties (e.g. the folding rate) is not straightforward. In order to address this issue, Mallam and Jackson developed a novel methodology (based on existing pulse-proteolysis experiments), which they combined with a coupled in vitro (i.e. cell free) transcription–translation system to determine the folding rates of nascent chains once they are synthesized by the ribosome [89]. In their first application of the method it became clear that proteins YibK and YbeA are able to fold spontaneously to their native states starting from fully unknotted conformations. However, the process is up to 1.5 orders of magnitude slower than refolding starting from chemically denatured (yet knotted) conformations [89], which may be taken as an indication that knotting is the rate-limiting step. In line with this hypothesis we observed in lattice simulations that when folding starts from partially folded conformations (*Q* = 0.3) that keeps 5 of the 12 interactions establishing within the knotted core, the folding rate is orders of magnitude larger than folding starting from unknotted or incorrectly knotted conformations [26].

Furthermore, Mallam and Jackson also observed that the folding rate of YibK and YbeA is significantly accelerated by the GroEL–GroES chaperonin complex, suggesting that a chaperonin-catalyzed knotting is likely to dominate in vivo [89]. The GroEL–GroES system contains a cylindrical chamber with a diameter of 45–70 Å that can accommodate proteins of size up to 60 KDa (i.e. with a chain length up to 550 amino acids) [100]; it is an ATP-driven molecular machine whose function is to increase folding efficiency (e.g. by fixing the structure of misfolded proteins and prevent aggregation) by sequestering protein in the confined environment of its chamber where refolding to the native state is allowed to take place in a series of ATP-driven cycles [101]. The exact details of how the chaperonin cage induces refolding to the correct native structure remain elusive and two models have been proposed for the mechanism according to which GroEL acts to enhance the yield of correctly folded proteins. In one of them, the passive-cage (also known as the Anfinsen's cage) hypothesis, the chaperonin does not actively influence folding; it only provides a restricted environment for refolding to occur via steric confinement. Steric confinement leads to a thermodynamic destabilization of the denatured state, which decreases the activation energy when folding is thermodynamically two-state [102]. In the iterative annealing model, on the other hand, the repeated binding and unbinding of the protein to the chaperonin cage are crucial for the protein to achieve the native state via denaturation of misfolded conformations [103,104]. Although the chaperonin mechanism in YibK and YibA has not been established, preliminary results suggest that it should not be limited to steric confinement and it has been proposed that the chaperonin facilitates unfolding of kinetically and topologically trapped intermediates or it stabilizes interactions that promote knotting [86].

**Fig. 7:**
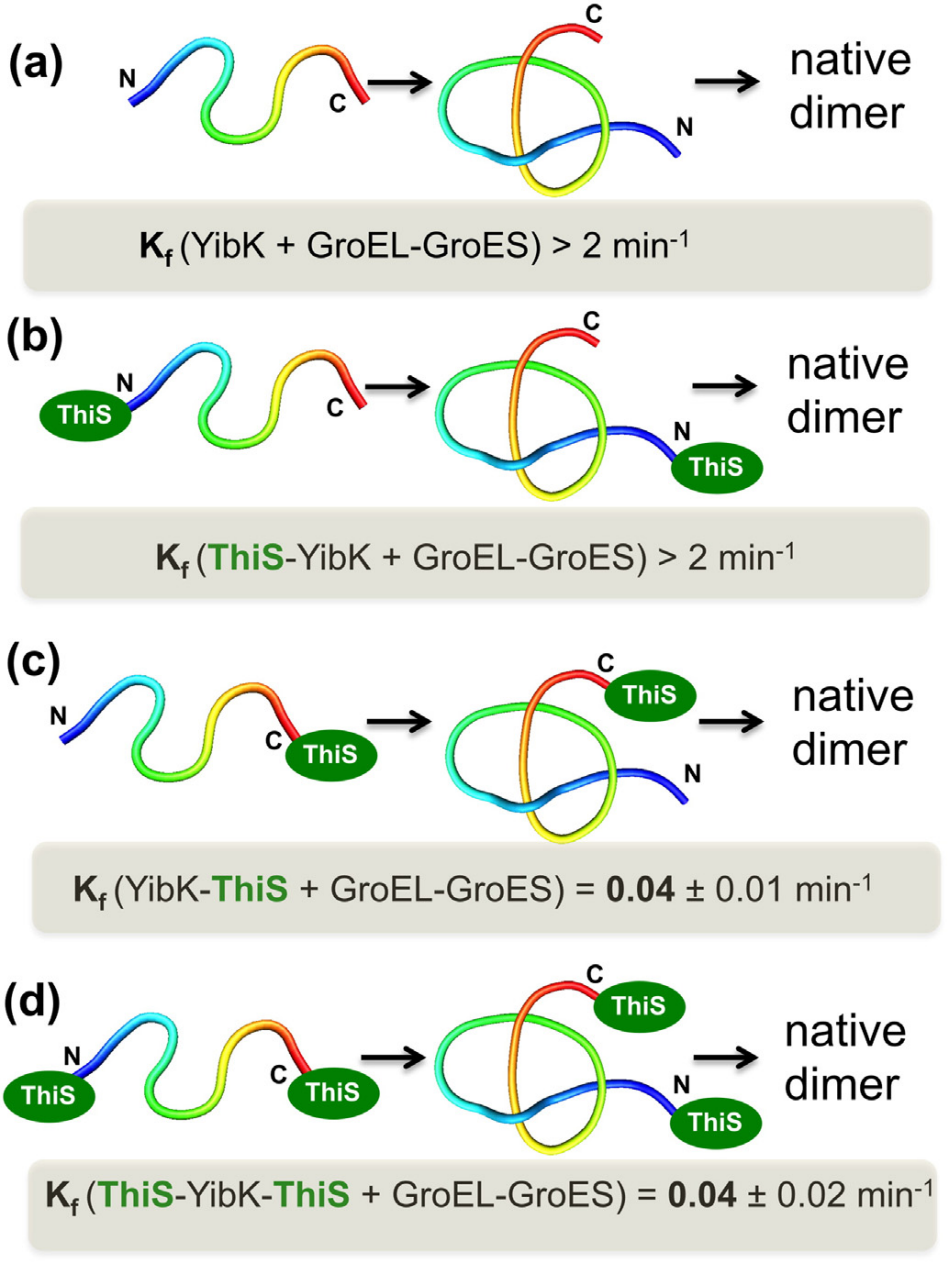
The folding rate of protein YibK (a) and YibK fused at one (b,c) or both termini (d) with ThiS. ThiS is a highly stable 91 residue domain (from thermophilic protein from *Archaeoglobus fugidus*) that hinders threading movements when fused to the chain ends. The measurement of the folding rate indicates that the folding mechanism of this knotted trefoil is based on a threading movement of the C-terminus. Indeed, there is only a drastic decrease of the folding rate when ThiS is fused to the carboxy-terminus. Similar results were reported for YibA (Figure adapted from [86]). Protein chains were prepared with knotplot (http://www.knotplot.com/).

More recently, Jackson's in vivo approach to protein folding was used to explore the folding mechanism of proteins YibK and YibA with fused stable domains at the C-terminus, N-terminus or both termini [86] (Fig. 7). The measurement of the folding rate shows very clearly that threading occurs via a mechanism in which the C-terminal end of the chain (which is the shortest knot tail) passes through a loop to form the knot, in strong agreement with predictions from molecular simulations (Fig. 3). Since co-translational folding is the folding process that occurs during protein synthesis, proceeding vectorially from the N-terminus to the C-terminus, these results also indicate that in vivo folding of these particular knotted proteins cannot occur co-translationally. It remains to be experimentally elucidated which exact conformation the C-terminus adopts during the knotting step, which will allow to establish the relative importance of a knotting mechanism based on slipknotting.

### 1.7 The role of knots in proteins

The motto ‘function follows form’ is a basic principle of biology operating at any hierarchical level of living matter. In particular, at the microscopic level of macromolecules, it specifically means that the function of a protein is determined by its three-dimensional native structure. Thus, the realization that deeply knotted proteins exist [7] immediately triggered the challenge of understanding the role of knots in proteins. Furthermore, the finding that knotted motifs can be conserved across different families (despite very low sequence similarity [14]), and that the complex and slow folding process of (deeply) knotted proteins is likely to be disadvantageous to their host organisms [105], has recently stimulated the quest for understanding the functional role of knots in proteins. Do knots convey an added structural or functional advantage to their carriers to compensate for their slow folding? Or are they simply a topological nuisance? If this is the case, why are these peculiar proteins evolutionarily conserved? How did they withstand evolutionary pressure?

Based on the analysis of specific knotted systems it has been suggested that knots (and slipknots) could play a role against degradation by sterically precluding translocation through the proteasome pore [12], provide structural stability in transporter proteins [14], enhance the structural rigidity of the native state [18], help shape and form the binding site of enzymes [106–108], enhance thermal [10,109] and mechanical [109–111] stability, or even alter enzymatic activity [110]. Knotted proteins have also been used as the building blocks of highly stable polymer filaments [94]. However, it has been recently pointed out that in the majority of cases it is not possible to determine the structural and/or functional advantages of knotted folds [14]. Therefore, one cannot rule out the possibility that in most of the times they do not exhibit any advantage at all. It may just be the case that knotted proteins have withstood evolutionary pressure because their folding process is—and has always been—assisted by chaperonins, which may have arisen very early during the evolution of densely crowded cells as a way to minimize protein aggregation [112].

As pointed out by Yeates, one experimental challenge in investigating the role of protein knots is constructing control systems that make it possible to directly access the effects of topology on folding properties [94]. While this can be achieved in real world models [91] and in off-lattice models [85] it is remarkably easier to prepare control structures in the context of lattice models. We have taken advantage of this possibility to systematically investigate the effects of knots (including knot depth, knot type and structural motif of the threading terminus) in thermal and kinetic stability. We found that knots in lattices (including deep ones) do not increase thermal stability (as measured by *T_m_*) but dramatically enhance kinetic stability (as measured by the unfolding rate) [18,26]. An enhanced kinetic stability for knotted proteins was also observed in off-lattice MD simulations [109]. Furthermore, kinetic stability of lattice proteins increases with the complexity of knot type (Fig. 6b), and can be enhanced even more by mutating native interactions that establish between residues located on the knotted core if these interactions are also major stabilizers of the transition state [18] in two-state folders. Interestingly, if the native conformation of the threading terminus is a hairpin docked onto the protein surface, the increase in kinetic stability of a lattice trefoil is even higher than that provided by a deep knot, specially when the temperature favors unfolding [26]. The conformation adopted by the termini in the native structure appears to be an important determinant of kinetic stability, being possibly more effective than knot depth at high temperature.

Kinetic stability is a property of the native state that is essential to maintain the biological function of the protein during a physiologically relevant timescale [113]. For this reason kinetic stability may represent a functional advantage. An enhanced kinetic stability may be particularly advantageous for proteins forming transmembrane channels since they are subjected to mechanical stress. Since knotted patterns appear to be preferentially conserved among transmembrane channels [14] we propose that the *systematic* functional role of knots in proteins is precisely that of enhancing their kinetic stability.

### 1.8 Summary and outlook

We discussed a selection of results on the folding of knotted proteins, including our own, that help building a picture of the current state-of-the-art in the field. Model systems investigated so far, both in simulations and in vitro experiments, highlight a remarkably choreographed process, populated by intermediate states, prone to topological trapping that is likely to be assisted in vivo by molecular chaperones due to slow folding rates. But an efficient and fast folding process should be an important driver of protein evolution because it contributes to increase the amount of functional protein that is available to the cell and decreases the population of intermediate states, thus avoiding pathological aggregation. In line with these arguments, a recent investigation showed that there is a clear overall increase in folding speed during evolution [114].

While aggregation-prone intermediates have not yet been detected in the folding of knotted proteins, one cannot rule out the possibility that they may be relevant species of their folding space. In this regard it would be interesting to explore if there is any relation between the degree of aggregation propensity [115] and knot type within the ensemble of knotted proteins found in the PDB. We conjecture that from a biological point of view knotted proteins are statistically rare because their slower folding speeds make them less prone to be evolutionary selected. On the other hand, the fact that folding speed is likely to decrease as the topological “complexity” of the embedded knot increases, may partially explain the relative abundance of the different knot types found in the PDB. One can argue that a slow folding speed poses no evolutionary caveat given the existence of chaperonins. However, it remains to be proved that chaperonins, and chaperones in general, have arisen very early during the evolution of densely crowded cells in order to assist protein folding [112].

Detailed microscopic pictures conveyed by simulations indicate that depending on the degree of participation of non-native interactions in protein energetics, the threading step upon which the chain becomes knotted is dominated by a slipknotted conformation, or by a direct threading movement of the smallest knot tail. Future studies, exploring an enlarged set of knotted proteins should be carried out in order to determine the generality of these mechanistic aspects and their relative importance. Furthermore, it is important to determine up to which extent the mechanistic details of the knotting step depends on fundamental polymeric properties of proteins (such as chain length) as suggested by simulations with homopolymers.

An interesting question, which has received little attention so far, concerns the overlap between the folding and knotting mechanisms in proteins with a two-state folding transition [18,85]. Assuming that there is evolutionary control of the folding speed, it should have resulted into additional pressure applied on the folding nucleus [116]. Therefore, an overlap between folding and knotting may imply that the interactions that nucleate the knot have also been optimized for folding speed (i.e. the optimization of the knotting mechanism is a side effect of folding optimization). In line with this conjecture, designed protein 2ouf, embedding a trefoil knot, folds with a two-state transition (with the nucleation of the transition state and nucleation of the knot being concomitant processes), and with fast knotting speed [85]. Exploring the relation between knotting and folding further may shed light on the evolution of knotted proteins.

The interplay between simulations and experiments should prove particularly fruitful to elucidate the mechanism according to which the chaperonin accelerates the folding of knotted proteins. One possibility, which we are currently investigating, is that the chaperonin transiently hampers the formation of short-range native interactions leading to local order, which physically hinders knotting in protein systems.

The biological role of knots in proteins is far from being understood. In particular, it is important to know if knots convey a systematic added functional advantage, such as an enhanced kinetic stability. Since many knotted proteins are enzymes it would be interesting to explore in a systematic way what is the role of knots in enzymes and, in particular, to understand how they influence and shape the catalytic center of the enzymes. If knots in proteins are only a folding nuisance, why are there knots in proteins? Why were they formed for the first time? Is there an optimal amino acid alphabet for knotting? One possibility is that knots could form more easily in primitive proteins, and were evolutionary conserved because, although they decrease folding speed, they also increase the kinetic stability of their carriers. Investigating these issues deeply in future studies may open new vistas on the general principles of protein evolution.
